# Nanostructured Lipid Carrier-Based Delivery of Pioglitazone for Treatment of Type 2 Diabetes

**DOI:** 10.3389/fphar.2022.934156

**Published:** 2022-07-12

**Authors:** Umair Ilyas, Muhammad Asif, Minglian Wang, Reem Altaf, Hajra Zafar, Mirza Muhammad Faran Ashraf Baig, Ana Cláudia Paiva-Santos, Muhammad Abbas

**Affiliations:** ^1^ Riphah Institute of Pharmaceutical Sciences, Riphah International University, Islamabad, Pakistan; ^2^ Faculty of Environment and Life Science, Beijing University of Technology, Bejing, China; ^3^ Department of Pharmacy, Iqra University Islamabad Campus, Islamabad, Pakistan; ^4^ School of Pharmacy, Shanghai Jiao Tong University, Shanghai, China; ^5^ Laboratory of Biomedical Engineering for Novel Bio-Functional, and Pharmaceutical Nano-Materials, Prince Philip Dental Hospital, Faculty of Dentistry, The University of Hong Kong, Hong Kong, China; ^6^ Department of Pharmaceutical Technology, Faculty of Pharmacy, University of Coimbra, Coimbra, Portugal; ^7^ REQUIMTE/LAQV, Group of Pharmaceutical Technology, Faculty of Pharmacy, University of Coimbra, Coimbra, Portugal

**Keywords:** pioglitazone, poor aqueous solubility, NLCs, nanoparticles, diabetes

## Abstract

Pioglitazone (PGZ) is utilized as a therapeutic agent in the management of (type 2) diabetes to control blood glucose levels. The existing research work was intended to make and optimize PGZ-containing NLCs (nanostructured lipid carriers). The fabricated nanostructured lipid carrier preparation was optimized by using different concentrations of the surfactants (Tween 80 and Span 80) and solid lipid (Compritol^®^ 888 ATO) and liquid lipid (Labrasol^®^) while keeping the concentration of drug (PGZ), and co-surfactants (poloxamer 188) the same. The optimized NLC formulation (PGZ-NLCs) was further assessed for physical and chemical characterization, *in vitro* PGZ release, and stability studies. The optimized PGZ-NLCs have shown an average diameter of 150.4 nm, EE of 92.53%, PDI value of 0.076, and zeta-potential of −29.1 mV, correspondingly. The DSC thermal analysis and XRD diffractograms had not presented the spectrum of PGZ, confirming the comprehensive encapsulation of PGZ in the lipid core. PGZ-NLCs showed significantly extended release (51% in 24 h) compared to the unformulated PGZ. Our study findings confirmed that PGZ-NLCs can be a promising drug delivery system for the treatment of type 2 diabetes.

## 1 Introduction

Pioglitazone (PGZ) is an oral therapeutic agent used in therapy of diabetes mellitus (DM). It is a thiazolidinedione derivative. The structural formula of PGZ is 5-[4-[2-(5-ethyl-2-pyridinyl) ethoxy] benzyl] thiazolidine- 2,4-dione (Waugh et al., 2006; Souri et al., 2008). In diabetic patients, insulin resistance is improved by the use of PGZ. It also reduces the macrovascular risks associated with diabetes (Mosure et al., 2019). PGZ significantly decreases the blood glucose levels in fasting and postprandial state and also reduces the glycosylated hemoglobin, while the beta-cell function is improved by its use (Shaveta et al., 2020). PGZ mainly performs its action by binding to peroxisome proliferator-activated gamma receptors (PPARs) (Smith, 2001). Stimulation of PPARs regulates the transcription of genes which controls the release of insulin responsible for the balancing of the production of glucose, glucose uptake, transport, and consumption in the organs. PGZ improves the sensitivity of the tissue to insulin and reduces gluconeogenesis which leads to improved glycemic control and decreases insulin resistance. PGZ being a member of the Biopharmaceutics Classification System (BCS) Class II exhibits low water solubility (0.00442 mg/ml) and high permeability. The half-life of PGZ is also very short (3–6 h). The low aqueous solubility of the PGZ corresponds to low dissolution. Poor solubility and decreased dissolution rate reduce the drug absorption and impart a negative effect on blood levels of the drug leading to decrease pharmacological activity (Elbary et al., 2008). Therefore, to achieve minimum therapeutic level concentration an increased dose of PGZ is required which can cause severe adverse effects (Shaveta et al., 2020). Furthermore, PGZ also undergoes metabolism, and many metabolites are produced in the liver to activate and inactivate metabolites by the process of oxidation and hydroxylation (Eckland and Danhof, 2000). The absorption of PGZ from GIT is further delayed in the presence of food (Pandey and Kohli, 2017). Keeping in view all these shortcomings associated with PGZ there is a necessity to develop effectual delivery systems of PGZ (Bhosale et al., 2016).

A number of approaches have been employed to deal with low water solubility constraints. Physicochemical modifications in the drug molecules are the major strategies among other approaches to improve solubility and enhance the surface area and drug release rate of drug particles. Lipidic drug delivery systems and solid dispersions are also among the solubility enhancement techniques through physical modifications of the system (Sinha et al., 2013; Malamatari et al., 2018). The bioavailability of the drugs given orally with poor solubility can also be enhanced through the latest developments in particle size modification techniques. The bioavailability of these drugs mainly depends upon the dissolution rate. It has been observed that the solubility is increased when the particle size is reduced. Improved solubility results in a higher dissolution rate. As a result, the drug’s bioavailability increases (Williams et al., 2013; Koradia et al., 2018). The equation of Noyes–Whitney explains the association between the dissolution velocity and the real surface area of the particles of the drug. The reduction of larger particles into smaller ones can cause a surge in the surface area and increased rate of dissolution (Pawar et al., 2014).

NLCs are taken to be the analogs of oil-in-water (o/w) emulsion as they are similarly formed as the o/w emulsion. The difference only takes place in the replacement of the oily phase of the emulsion for solid lipids in the presence of liquid lipids. Accordingly, NLCs may be comprised of a lipid blend of solid and liquid lipid distributed in a water phase at elevated temperatures. Sometimes, a surfactant or mixture of surfactants and co-surfactant is used to stabilize the formulation. NLCs have a particle size in the range of 50–1,000 nm having a spherical shape (Junghanns and Müller, 2008). NLCs have been utilized as an alternate drug carrier system to other colloidal systems of drug delivery. NLCs are comprised of solid lipids, liquid lipids, surfactants and/or cosurfactants, and water. A distinctive solid type lipid that is employed in these kinds of carrier systems should have the ability to melt above the body temperature of 37°C.

NLCs have exhibited many advantages over other colloidal drug delivery systems and simultaneously minimized the problems related with other colloidal carrier systems (Mehnert and Mäder, 2012). Generally, a solid core in NLCs offers various advantages in the presence of a liquid core. Usually, liposomes and emulsions also fail to protect the encapsulated drug. and a burst release of drug can happen from emulsions or uncontrolled release of drug from the liposomal formulation. NLCs exhibit controlled release effect, at the same time also protects the drug from degradations. NLCs have better stability and high capability to load the drug. NLCs also need lower quantities of organic solvents in production, which declines the chances of toxicity. In conclusion, by comparison with other colloidal nanoparticles, NLC production procedures are cost-effective and are easily scalable (Junghanns and Müller, 2008).

The aim of this research work was to develop and assess pioglitazone-loaded NLCs for better drug delivery and enhanced solubility that can be used to improve the antidiabetic potential of pioglitazone.

## 2 Materials and Methods

### 2.1 Materials

Pioglitazone hydrochloride (PGZ) drug was received as a gift sample from Xellia Pharmaceuticals. Methanol, Tween 80, poloxamer 188, Span 80, Compritol^®^ 888 ATO, and Labrasol^®^ were procured from Sigma-Aldrich (St. Louis, MO, United States). Analytical-grade quality was assured for all chemicals.

### 2.2 Preparation of Pioglitazone–Nanostructured Lipid Carriers

PGZ-NLCs were formulated by nano-emulsion template technique with minor changes. In brief, the blend of PGZ, Tween 80, Span 80, Compritol^®^ 888 ATO, Labrasol^®^, and poloxamer 188 were put in a water bath and melted at 65°C. Deionized water was filtered and then heated up to 65°C. A volume of 5 ml of warmed deionized water was added to the melted blend of lipids and surfactants with constant stirring at a rotation speed of 750 rpm for 30 min. A transparent nano-emulsion was obtained. The temperature of the system was sustained at 70°C throughout the making of nano-emulsion. Afterward, the warm nano-emulsion was quickly cooled down at a temperature below 4°C in the ice container along with constant stirring at 750 rpm to solidify the lipids to produce PGZ-NLCs. Free PGZ and large aggregates from the PGZ-NLC formulation were withdrawn by filtering it through a 0.45-pm syringe filter. For further study, PGZ-NLCs were held in reserve at 4°C (Rizvi et al., 2019).

### 2.3 Optimization of Pioglitazone–Nanostructured Lipid Carriers

PGZ-NLC formulations were optimized for different Tween 80 and Span 80 concentrations in the surfactant blend, and their influence on mean diameter of the particle, EE, PDI, and zeta potential was determined. The amount of PGZ and surfactant component poloxamer 188 was kept constant, while the concentration of solid lipid Compritol^®^ 888 ATO and liquid lipid Labrasol was varied. Table 1 contains the concentration of all the formulation components used.

**TABLE 1:** Composition of all formulations.

Formulation code	PGZ	Compritol 888 ATO	Labrasol	Tween 80	Span 80	Poloxamer 188
F1	5	5	5	77	23	20
F2	5	4	6	78	22	20
F3	5	7	3	79	21	20
F4	5	8	2	85	15	20
F5	5	9	1	92	9	20

### 2.4 Characterization of Pioglitazone–Nanostructured Lipid Carriers

#### 2.4.1 Particle Size, Size Distribution, and Zeta Potential Analysis

Mean particle size, PDI (polydispersity index) value, and zeta potential of optimized PGZ-NLCs were determined using a Zetasizer ZS 90 (Malvern Instruments, Malvern, Worcestershire, United Kingdom). For analysis, PGZ-NLCs were suitably diluted with deionized water.

#### 2.4.2 Encapsulation Efficiency

The amount of PGZ encapsulated in PGZ-NLCs was determined by using a UV-visible spectrophotometer. Before carrying out analysis, PGZ in free form and aggregates of larger size were removed by passing the NLC formulation through a syringe filter of 0.45 p.m. The filtered PGZ-NLCs were dissolved in methanol. Analysis for PGZ contents was carried out by using UV-visible spectrophotometer at 220 nm (V-530; JASCO Corporation, Tokyo, Japan) (Shaveta et al., 2020). The encapsulation efficiency and drug loading (%) of PGZ-NLCs are assessed by using the equations given as follows:

Encapsulation efficiency (%) = PGZ amount in PGZ-NLCs/PGZ total amount added×100,

Drug Loading (%) = PGZ amount in PGZ-NLCs/total weight of PGZ - NLCs×100.

#### 2.4.3 Morphology Analysis

The morphology of PGZ-NLCs was examined using scanning electron microscopy (SEM) (Hitachi S-4100, Hitachi Ltd., Tokyo, Japan). A minute quantity of lyophilized PGZ-NLCs was spread on a carbon-coated tape and dried at room temperature. A thin layer of gold was used to sputter the sample under vacuum (Rizvi et al., 2019).

#### 2.4.4 FTIR Analysis

To inspect the PGZ compatibility with excipients, FTIR spectra of unformulated PGZ, Compritol^®^ 888 ATO, poloxamer 188, and lyophilized PGZ-NLCs were attained by means of an FTIR spectrophotometer (Eco Alpha II- Bruker, Billerica, MA, United States). The infra-red spectrum was gained in the range of 4,000–400 cm^−1^. Lyophilization of PGZ-NLCs was executed using a freeze-dryer (TFD5503, IlShin BioBase Co., Ltd. Gyeonggido, Republic of Korea) (Pireddu et al., 2016; Casula et al., 2021).

#### 2.4.5 Powdered X-Ray Diffractometry

The powder x-ray diffraction examination of lyophilized PGZ-NLCs and their specific solid constituents (unformulated PGZ, Compritol^®^ 888 ATO, and poloxamer 188) was carried out using a powder X-ray diffractometer (D8 Advance-Bruker, Billerica, MA, United States). All the samples were scanned at an angle of 2θ in the range of 3–70°, and at 0.02°/s rotation with a current of 40 mA and 40 kV voltage (Rizvi et al., 2019).

#### 2.4.6 Differential Scanning Calorimetry

The thermal features of lyophilized PGZ-NLCs and their different solid ingredients (unformulated PGZ, Compritol^®^ 888 ATO, and poloxamer 188) were inspected using a differential scanning calorimeter (DSC Q20; TA Instrument, New Castle, DE, United States). For the DSC study, the sample was positioned in an aluminum pan and heated over a temperature range of 0–200°C at 10°C/min rate (Rizvi et al., 2019).

#### 2.4.7 *In Vitro* Release of Pioglitazone–Nanostructured Lipid Carriers

The *in vitro* release profile of PGZ-loaded NLCs was estimated via the dialysis bag method with simulated gastric fluid (SGF) of pH 1.2 and simulated intestinal fluid (SIF) of pH 6.8 as dissolution medium at 37 ± 0.5°C temperature and 100 rpm rotation. PGZ-NLC formulation equal to 5 mg of PGZ was put in a dialysis membrane of 3,500 Da molecular cut-off weight (Spectrum Laboratories, Inc., Rancho Dominguez, CA, United States). Before carrying out the release study, the dialysis membrane was submerged in SGF or SIF. 0.5%, w/v Tween 80 and 5%, v/v ethanol were put into the release media for maintaining the sink conditions. Samples of 2 ml were drawn out from release media at determined time intervals for 24 h. To sustain a persistent volume after sample removal, the release medium was instantly substituted with an equivalent volume of fresh fluid. The samples were analyzed for PGZ at 220 nm by using a UV-visible spectrophotometer (Rizvi et al., 2019).

#### 2.4.8 Statistical Analysis

All the experimentations were carried out in triplicate, and data were exhibited as mean ± S.D. SPSS software (SPSS Inc., Chicago, IL, United States) was utilized to analyze statistical significance amongst groups by employing Student’s t-test at the *p* < 0.05 significance level.

## 3 Results

### 3.1 Preparation of Pioglitazone–Nanostructured Lipid Carriers

PGZ-loaded NLCs were efficaciously fabricated by the nano-emulsion-based method with decent uniformity and reproducibility. Compritol^®^ 888 ATO was utilized as lipid solid for the formation of the outer solid lipid core of PGZ-NLCs, while Labrasol was utilized as liquid lipid to fill the imperfections in the solid core, and the prepared NLC formulation was made stable by using a mixture of Tween 80 as a surfactant and Span 80 and poloxamer as co-surfactant.

### 3.2 Optimization of Pioglitazone–Nanostructured Lipid Carrier Formulations

Table 2 exhibits the obtained results of various formulations of PGZ-NLCs optimized for different amounts of Tween 80 and Span 80 in the surfactant blend and their effect on particle diameter, EE, PDI, and zeta potential was determined. Based upon the features of particle size, PDI, EE, and zeta potential values, formulation 3 was carefully chosen as optimized and was further characterized for various physicochemical constraints.

**TABLE 2:** Experimental results of various PGZ-NLC formulations.

Formulation code	Particle size (nm)	PDI	EE %	Zeta potential (mV)
F1	319	0.28	95.03	17
F2	187.7	0.097	91.14	20.9
F3	150.4	0.076	92.53	−29.1
F4	124.6	0.36	93.21	21.5
F5	251.7	0.441	86.63	27.1

### 3.3 Characterization of EPL-NCs

#### 3.3.1 Particle Size and Size Distribution and Zeta Potential Analysis

**FIGURE 1:**
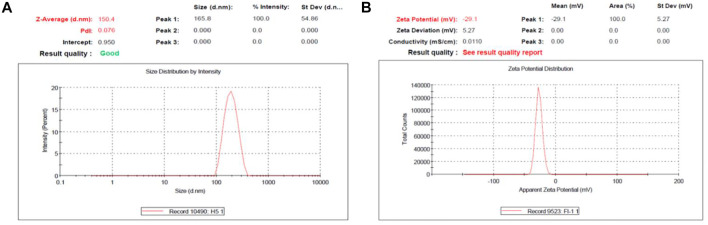
Optimized PGZ-NLCs. **(A)** Particle size distribution; **(B)** zeta potential.

PGZ-NLCs presented an average diameter of particles of 150.4 nm, a PDI value of 0.076 articulating thin size distribution, and a zeta potential value of −29.1 mV positively. Figure 1 displays the particle size distribution (A) and zeta potential (B) of the optimized PGZ-NLCs.

#### 3.3.2 Incorporation Efficiency

PGZ-NLCs showed a high drug encapsulation efficiency of 92.53%, and drug loading was found to be 6%.

#### 3.3.3 Morphology Analysis

**FIGURE 2:**
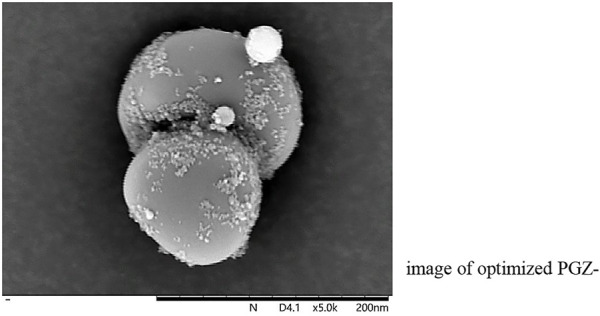
SEM image of optimized PGZ-NLCs.

The morphology and shape analysis of PGZ-NLCs was completed over SEM, and the SEM image in Figure 2 exposed that the shape of the PGZ-NLCs was spherical and the surfaces were smooth without any aggregation.

#### 3.3.4 FTIR Analysis

**FIGURE 3:**
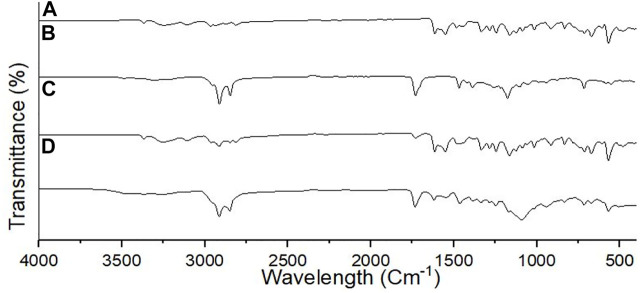
FTIR spectra of **(A)** PGZ, **(B)** Compritol 888 ATO, **(C)** poloxamer 188, and **(D)** optimized PGZ-NLCs.

The FTIR spectrum of unformulated PGZ, poloxamer 188, Compritol^®^ 888 ATO, and lyophilized PGZ-NLCs was carried out to assess the compatibility of the drug with the excipients. In Figure 3, the FTIR spectra of PGZ showed distinctive N–H stretch at 3,069 cm^−1^, a peak of C–H stretch at 2,985 cm^−1^, a peak of C= O stretching at 1732 cm^−1^, the C–C aromatic stretch at 1,601 cm^−1^, and the peak of C–S bond at 1,220 cm^−1^.

#### 3.3.5 Powdered X-Ray Diffraction Studies

**FIGURE 4:**
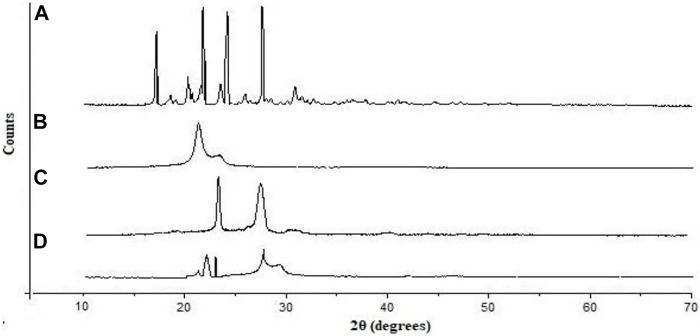
Powder X-ray diffraction pattern of **(A)** PGZ, **(B)** Compritol 888 ATO, **(C)** poloxamer 188, and **(D)** optimized PGZ-NLCs.

XRD spectra of unformulated PGZ, poloxamer 188, Compritol^®^ 888 ATO, and PGZ-NLCs were determined, and are displayed in Figure 4. The unformulated PGZ diffractogram presented intense distinguishing peaks of crystalline nature at 20 angles of 11.2, 15.8, 18.2, and 21.6. Compritol^®^ 888 ATO exhibited distinguishing diffraction peaks at 20 angles of 21.3 and 23.7. No characteristic peak of PGZ in the XRD spectrum of PGZ-NLCs was found, and the diffraction pattern of Compritol^®^ 888 ATO was merely observed at angles of 21.1 and 23.3 of 20 diffractions.

#### 3.3.6 Differential Scanning Calorimetry

**FIGURE 5:**
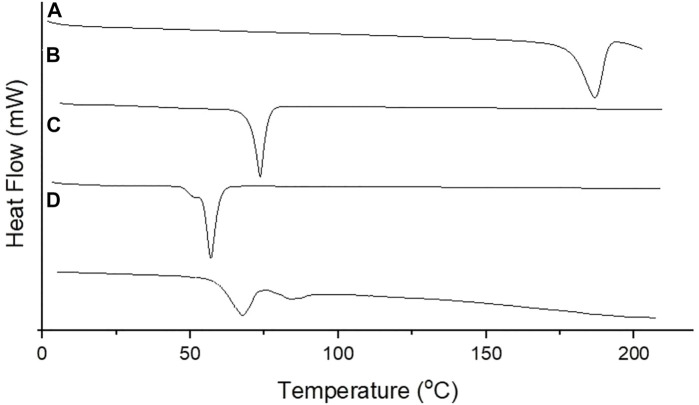
DSC thermogram of **(A)** PGZ, **(B)** Compritol 888 ATO, **(C)** poloxamer 188, and **(D)** optimized PGZ-NLCs.

Thermal analysis of PGZ, Compritol^®^ 888 ATO, poloxamer 188, and PGZ-NLCs was performed by DSC, and the obtained results are displayed in Figure 5. The thermal spectrum of PGZ showed a sharp endothermic peak at 184°C while Compritol^®^ 888 ATO at 74.6°C, which corresponded to the melting point of drug and lipid. The thermal spectrum of PGZ-NLCs did not show any endothermic melting peak of PGZ, and the thermal spectrum presented a Compritol^®^ 888 ATO melting peak at 66.2°C with a slight peak shifting to the lower temperature.

#### 3.3.7 *In Vitro* Release of Pioglitazone–Nanostructured Lipid Carriers

**FIGURE 6:**
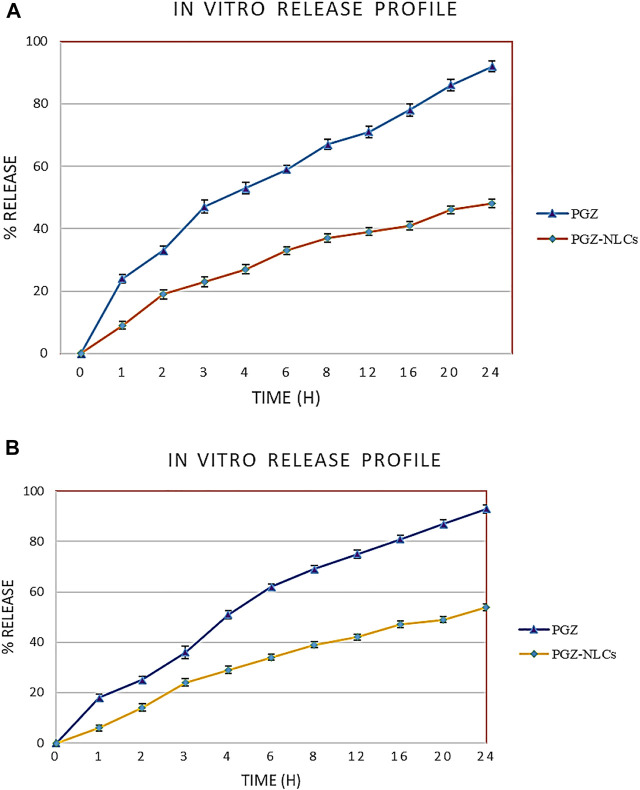
**(A)** Comparative profiles of *in vitro* release of unformulated PGZ and PGZ-NLCs in simulated gastric fluid. **(B)** Comparative profiles of *in vitro* release of unformulated PGZ and PGZ-NLCs in simulated intestinal fluid.

The *in vitro* drug release of PGZ-NLCs was compared with the release profile of unformulated PGZ. The assessment of drug release was made in simulated gastric fluid at pH 1.2 and simulated intestinal fluid having pH 6.8 at 37°C. Owing to the less aqueous solubility of PGZ, Tween 80 0.5%, w/v and ethanol 5%, v/v were incorporated into the release media to sustain sink conditions by dissolving the released PGZ. Figure 6 exhibits the results of *in vitro* release of PGZ-NLCs and unformulated PGZ in both media. PGZ-NLCs exhibited 19% of PGZ release in the early 2 h in simulated gastric fluid shadowed by slow and continual release form with a collective release of 51% within 24 h (Figure 6A). In differing to PGZ-NLCs, PGZ suspension displayed quicker release with 33% and 92% of PGZ released after a time of 2 and 24 h, correspondingly.

In simulated intestinal fluid, the % release of PGZ from PGZ-NLCs was ∼14% at 2 h equated to 25% from PGZ dispersal. Within 24 h, PGZ-NLCs and PGZ suspension presented 54% and 93% release of PGZ, respectively.

#### 3.3.8 Stability Study

**FIGURE 7:**
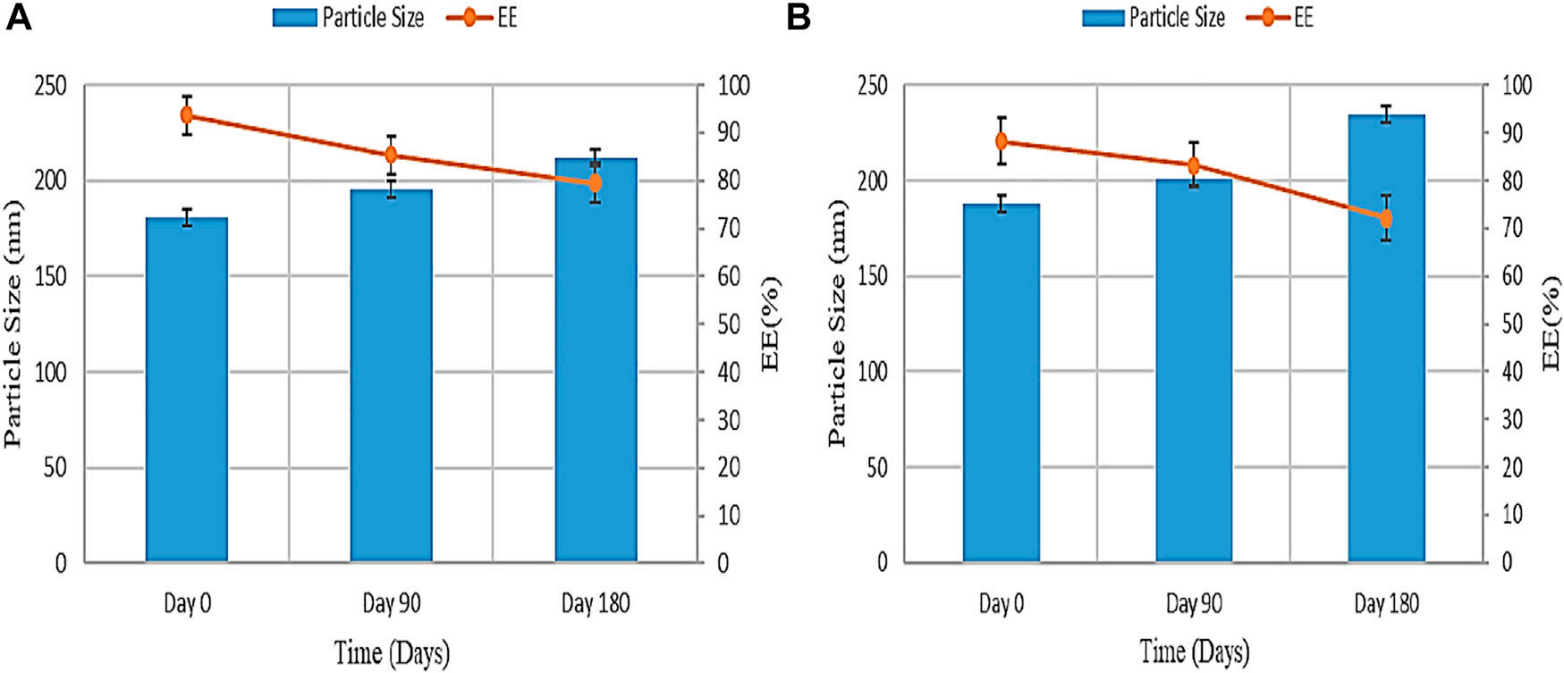
Stability study of PGZ-NLCs **(A)** at 25°C and **(B)** 40°C.

The result of the stability study of PGZ-NLCs at room temperature and accelerated state according to the ICH guiding principle are described in Figures 7A,B. Subsequently, storing the samples for 6 months, the samples were examined at specified intervals. A rise in particle size and drop in EE was found on storage at room temperature and accelerated state. The particle size and entrapment efficiency noted at the start of the study were 150.4 ± 4.33 nm and 92.53% ± 4.11%, and, later a period of 180 days, the particle size at 25°C and 60% RH was increased to 231.32 ± 2.13 nm and encapsulation efficiency was decreased to 76.73% ± 3.27%, correspondingly. The particle size and entrapment efficiency outcome for PGZ-NLCs kept at 40°C were found to be significantly changed compared to the PGZ-NLCs maintained under storage conditions of 25°C. The primary particle size and entrapment efficiency were found to be 150.4 ± 4.33 nm and 92.53% ± 4.78%, and after the period of 180 days, it exposed a significant rise in particle size (254.66 ± 5.32) and drop in entrapment efficiency (71.87% ± 3.41%).

## 4 Discussion

PGZ-loaded NLCs were efficaciously fabricated by the nano-template engineering method with decent uniformity and reproducibility. Compritol^®^ 888 ATO was utilized as solid lipids while Labrasol as liquid lipid for the preparation of the outer solid lipid core of PGZ-NLCs, and the prepared NLCs were stabilized by using a surfactant mixture of Tween 80, Span 80, and poloxamer. Compritol^®^ 888 ATO is a pharmaceutically suitable lipid having the characteristics of biocompatibility and biodegradability. The types of solid lipids which have a melting temperature higher than the temperature of the body own solid aquaphobic connections with lipotropic drugs which is the cause of high encapsulation efficiency of the drug and the sustained release of the drug due to stable outer core of the lipid. Tween 80 and Span 80, non-ionic surfactants, along with poloxamer 188, were utilized to form a surfactant mixture miscible with the lipid core. The selection of surfactants for the stabilization of the nano-emulsions also rests the HLB value (hydrophilic and lipophilic balance) of the surfactant-containing system. To get an optimal stability, the HLB value of the mixture of surfactants should be close to that essential for a specific lipid. The cumulative HLB of Tween 80, Span 80, and polaxamer 188 was 15.5, which is required to stabilize the Compritol^®^ 888 ATO lipid core. PGZ-NLCs were optimized for particle size, PDI value, and encapsulation efficiency (Rizvi et al., 2019).

The NLC particle size has a significant effect on the physical stability of the NLC formulation, drug release rate, and *in vivo* performance*.* Various parameters affect the particle size such as the type of the lipid, surfactant, and their properties, the technique used for the production, and conditions set for the processing (such as temperature, time, and number of cycles pressure). The mean particle size of the drug-loaded NLCs rises with the surge in the melting point of lipids. It has been proposed that larger particle sizes are due to an increase in the viscosity of the dispersion medium and a rise in the lipids' melting point. Furthermore, constraints such as lipid structure, crystallization rate, and size will differ individually with the type of lipid. The composition of the lipids also considerably affects the quality of the NLCs. A lipid content higher than 5%–10% causes an increase in particle size and PDI value due to augmented viscosity of the NLC dispersions which enhances the particle agglomeration rate (Mehnert and Mäder, 2001). In addition, properties of the surfactants and their concentration used in the formulation also affect the particle size and the effectiveness of the NLCs as a system of drug delivery. Small size particles increase the surface area of NLCs. According to the Ostwald ripening phenomenon, the enhanced surface area causes thermodynamical variability and can result in phase separation (Mehnert and Mäder, 2001). For this reason, the amount of the surfactant used should be adequate to cover all the afresh molded surfaces in NLC fabrication. Surfactants prevent the incidence of phase separation by dropping the interfacial tension among the lipid phase and the aqueous phases. Excess surfactant might be present in the formulation in various forms such as monomers, micelles, or liposomes. It has been observed that an NLC formulation stabilized by a mixture of surfactant and co-surfactant has a small particle size and improved stability as compared to the NLCs formed with a single surfactant. Siekmann and Westesen, (1996) described that 10% w/v tyloxapol was mandatory to stabilize a dispersion of 10% w/v tripalmitin. Established that an NLC formulation stabilized by an ionic surfactant exhibited smaller particle size as equated to an NLC suspension stabilized by a surfactant of nonionic nature.

The PGZ-loaded NLC particle size and PDI value were somewhat enlarged related to blank NLCs (133.2 nm and 0.201). The reason for this effect was the entrapment of PGZ in the lipid core (Qureshi et al., 2017). The particle size of NLCs also plays a vital part in the uptake of NLCs in GIT after oral administration. For an effective drug transport into the intestinal lymphatic system, a particle size below 300 nm is favorable (Estella-Hermoso de Mendoza et al., 2009). Obtained PGZ-NLCs had a uniform size distribution as demonstrated by a smaller PDI value (0.08) and a size distribution curve of unimodal nature. PDI value < 0.2 is normally recognized as the optimal value to specify consistent nanoparticle distribution (Zhang et al., 2020). Another vital constraint that influences the physical stability of the colloidal system is zeta potential which is the degree of net surface charge. High values for zeta potential produce stability to the dispersion systems of the colloids by avoiding particle aggregation owing to electrostatic repulsion among likewise charged nanoparticles. PGZ-NLCs presented a zeta potential value of −29.1 mV, which was not dissimilar to the blank NLCs significantly (24.9 mV). A zeta potential value between −20 and −30 mV or +20 and +30 mV is considered appropriate to guarantee electrostatic steadiness.

PGZ-NLCs revealed high drug encapsulation efficiency of 92.53% and drug loading was found to be 8% which is significant for dropping the net weightiness or size of the ultimate dosage form (Zeb et al., 2020). The surface morphology and shape analysis of PGZ-NLCs was completed over SEM, and the SEM image in exposed that the shape of the PGZ-NLCs was spherical and the surfaces were smooth without any aggregation.

The FTIR spectral analysis of unformulated PGZ, Compritol^®^ 888 ATO, poloxamer 188, and lyophilized PGZ-NLCs was carried out to assess the compatibility of the drug with the excipients. In the FTIR spectra exhibited that characteristic peaks of PGZ and Compritol 888 ATO were intact and also present in the PGZ-NLCs spectra. This result determined that no interaction took place between PGZ and formulation excipients and PGZ was compatible with all the components (Shaveta et al., 2020).

XRD spectra of pure PGZ, Compritol^®^ 888 ATO, poloxamer 188, and PGZ-NLCs were determined and are displayed. No characteristic peak of PGZ in the XRD spectrum of PGZ-NLCs was found, and only the diffraction peaks of Compritol^®^ 888 ATO were observed This result can be detected due to the nanosized range of NLCs and solubilization and encapsulation of PGZ in the lipid core as well as the change of crystalline PGZ to amorphic form in PGZ-NLCs (AmeeduzzafarEl-Bagory et al., 2019).

The DSC analysis of PGZ, Compritol^®^ 888 ATO, poloxamer 188, and PGZ-NLCs was performed, and the obtained results are shown. The thermal spectrum of PGZ showed a sharp endothermic peak at 184°C while Compritol^®^ 888 ATO at 74.6°C, which corresponded to the melting point of drug and lipid. The thermal spectrum of PGZ-NLCs did not show any endothermic melting peak of PGZ, and the thermal spectrum presented a Compritol^®^ 888 ATO melting peak at 66.2°C with a little peak shifting to the lower temperature. This shifting of the Compritol^®^ 888 ATO melting peak to the lower temperature in PGZ-NLCs thermal analysis is due to the drop in the particle size. For melting big crystals, long time and more energy are required, and a decline in size enhances the particle surface area which leads to a drop in the melt temperature in comparison to Compritol^®^ 888 ATO. This behavior can also be owing to the Compritol^®^ 888 ATO dispersed state and surfactants presence (Span 80, Tween 80, and poloxamer 188). The nonappearance of PGZ peaks in the PGZ-loaded NLC spectrum specifies the complete encapsulation of PGZ in the lipid matrix of Compritol^®^ 888 ATO because of the amorphization. The obtained findings were in agreement with the earlier publication (Trevaskis et al., 2015; Rizvi et al., 2019). It is notable to indicate that the alteration of crystalline PGZ to amorphous form in PGZ-NLCs may be the reason behind the enhanced solubility and improved bioavailability.

Exhibits the *in vitro* profile of PGZ-NLCs and unformulated PGZ in both media. PGZ-NLCs exhibited 19% release of PGZ in the early 2 h in simulated gastric fluid shadowed by slow and continual release form in a collective release of ∼51% within 24 h (Figure 6A). In contrast to PGZ-NLCs, PGZ suspension displayed quicker release with 33% and 92% of PGZ released after a time of 2 and 24 h, respectively. In simulated intestinal fluid, the % release of PGZ from PGZ-NLCs was ∼14% at 2 h equated to 25% from PGZ dispersal (Figure 6B). Within 24 h, PGZ-NLCs and PGZ suspension presented 54% and 93% release of PGZ, respectively. To some extent, the quicker release of PGZ from PGZ-NLCs in the early 2 h may be due to the fast release of a minor portion of PGZ stuck at the surface of NLCs. The preliminary increased release rates are due to this drug present on the outer surface which releases rapidly in release media. Afterward, PGZ-NLCs showed a sluggish, sustained, and partial drug liberation. The effect could be described by the slow diffusion of PGZ which was encapsulated in the lipid core of PGZ-NLCs owing to sturdy drug–lipid interactions and the steady attrition of the lipid matrix. The PGZ-NLC profile showing sustained release effect recommends a drug-enriched core model for the encapsulation of PGZ in NLCs. Though PGZ-NLCs presented somewhat quicker release first, a significant burst release effect was not detected which is an indication of drug-enriched core shell model (Rizvi et al., 2019).

The result of the stability study of PGZ-NLCs at room temperature and accelerated state according to the ICH guiding principle are described. Subsequently, storing the samples for 6 months, the samples were examined at specific time intervals. A rise in particle size and drop in EE was found on storage at room temperature and accelerated state. The particle size and entrapment efficiency noted at the start of the study were 180.6 ± 4.33 nm and 92.53% ± 4.11%, and, later a period of 180 days, the particle size at 25°C and 60% RH was increased to 211.56 ± 3.53 nm and encapsulation efficiency was decreased to 79.51% ± 4.87%, correspondingly. The particle size and entrapment efficiency outcome for PGZ-NLCs kept at 40°C were found to be significantly changed compared to the PGZ-NLCs maintained under storage conditions of 25°C. The primary particle size and entrapment efficiency were found to be 187.86 ± 3.21 nm and 88.31% ± 4.78%, and, after the period of 180 days, it exposed a significant rise in particle size (234.78 ± 7.14) and drop in entrapment efficiency (72.19% ± 4.14%). These differences in the outcomes were found due to the particle agglomeration and drug leakage from NLCs at an increased temperature and similarly may be because of the instability at the high temperature upon longer augmented storage conditions.

## 5 Conclusion

In this study, the successful development of nanostructured lipid carriers was achieved by the nano-template engineering technique. Pioglitazone was successfully incorporated into the fabricated NLCs by the temperature-controlled solidification procedure. The developed method was very simple. It was reproducible, and the NLCs were prepared without the use of any organic solvent. No sophisticated apparatuses and instruments were required, and the developed method has the potential to scale up easily for any larger-scale manufacture. The optimized PGZ-NLCs have shown a mean particle size of 150.4 nm, EE of 92.53%, PDI value of 0.076, and zeta potential of −29.1 mV, correspondingly. The DSC thermal analysis and XRD diffractograms did not present any peak of PGZ confirming the comprehensive entrapment of PGZ in the lipid core. PGZ-NLCs showed significantly extended release (51% in 24 h) compared to the unformulated PGZ. Our study findings revealed that PGZ-NLCs could be a potential drug delivery method for the treatment and management of type-2 diabetes.

Future work of this study comprises examining the consequence of pH on the *in vitro* drug release of PGZ from PGZ-NLCs and *in vitro* cell toxicity studies and *in vivo* antidiabetic studies.

## Data Availability

The original contributions presented in the study are included in the article/Supplementary Material; further inquiries can be directed to the corresponding authors.

## References

[B1] AmeeduzzafarEl-Bagory I. El-Bagory I. Alruwaili N. K. Elkomy M. H. Ahmad J. Afzal M. (2019). Development of Novel Dapagliflozin Loaded Solid Self-Nanoemulsifying Oral Delivery System: Physiochemical Characterization and *In Vivo* Antidiabetic Activity. J. Drug Deliv. Sci. Technol. 54, 101279. 10.1016/j.jddst.2019.101279

[B2] Bhosale U. Galgatte U. Chaudhari P. (2016). Development of Pioglitazone Hydrochloride Lipospheres by Melt Dispersion Technique: Optimization and Evaluation. J. App Pharm. Sci. 6 (01), 107–117. 10.7324/japs.2016.600118

[B3] Casula L. Sinico C. Valenti D. Pini E. Pireddu R. Schlich M. (2021). Delivery of Beclomethasone Dipropionate Nanosuspensions with an Electronic Cigarette. Int. J. Pharm. 596, 120293. 10.1016/j.ijpharm.2021.120293 33497704

[B4] Eckland D. Danhof M. (2000). Clinical Pharmacokinetics of Pioglitazone. Exp. Clin. Endocrinol. diabetes 108 (Suppl. 2), 234–242. 10.1055/s-2000-8525

[B5] Elbary A. A. Kassem M. A. Abou Samra M. M. Khalil R. M. (2008). Formulation and Hypoglycemic Activity of Pioglitazone-Cyclodextrin Inclusion Complexes. Drug Discov. Ther. 2 (2), 94–107. 22504505

[B6] Estella-Hermoso de Mendoza A. Campanero M. A. Mollinedo F. Blanco-Prieto M. J. (2009). Lipid Nanomedicines for Anticancer Drug Therapy. J. Biomed. Nanotechnol. 5 (4), 323–343. 10.1166/jbn.2009.1042 20055079

[B7] Junghanns J. U. Müller R. H. (2008). Nanocrystal Technology, Drug Delivery and Clinical Applications. Int. J. Nanomedicine 3 (3), 295–309. 10.2147/ijn.s595 18990939 PMC2626933

[B8] Koradia K. D. Parikh R. H. Koradia H. D. (2018). Albendazole Nanocrystals: Optimization, Spectroscopic, Thermal and Anthelmintic Studies. J. drug Deliv. Sci. Technol. 43, 369–378. 10.1016/j.jddst.2017.11.003

[B9] Malamatari M. Taylor K. M. G. Malamataris S. Douroumis D. Kachrimanis K. (2018). Pharmaceutical Nanocrystals: Production by Wet Milling and Applications. Drug Discov. Today 23 (3), 534–547. 10.1016/j.drudis.2018.01.016 29326082

[B10] Mehnert W. Mäder K. (2001). Solid Lipid Nanoparticles: Production, Characterization and Applications. Adv. Drug Deliv. Rev. 47 (2), 165–196. 10.1016/s0169-409x(01)00105-3 11311991

[B11] Mehnert W. Mäder K. (2012). Solid Lipid Nanoparticles: Production, Characterization and Applications. Adv. drug Deliv. Rev. 64, 83–101. 10.1016/j.addr.2012.09.021 11311991

[B12] Mosure S. A. Shang J. Eberhardt J. Brust R. Zheng J. Griffin P. R. (2019). Structural Basis of Altered Potency and Efficacy Displayed by a Major *In Vivo* Metabolite of the Antidiabetic PPARγ Drug Pioglitazone. J. Med. Chem. 62 (4), 2008–2023. 10.1021/acs.jmedchem.8b01573 30676741 PMC6898968

[B13] Pandey V. Kohli S. (2017). SMEDDS of Pioglitazone: Formulation, *In-Vitro* Evaluation and Stability Studies. Future J. Pharm. Sci. 3, 53–59. 10.1016/j.fjps.2017.02.003

[B14] Pawar V. K. Singh Y. Meher J. G. Gupta S. Chourasia M. K. (2014). Engineered Nanocrystal Technology: *In-Vivo* Fate, Targeting and Applications in Drug Delivery. J. Control Release 183, 51–66. 10.1016/j.jconrel.2014.03.030 24667572

[B15] Pireddu R. Caddeo C. Valenti D. Marongiu F. Scano A. Ennas G. (2016). Diclofenac Acid Nanocrystals as an Effective Strategy to Reduce *In Vivo* Skin Inflammation by Improving Dermal Drug Bioavailability. Colloids Surf. B Biointerfaces 143, 64–70. 10.1016/j.colsurfb.2016.03.026 26998867

[B16] Qureshi O. S. Kim H. S. Zeb A. Choi J. S. Kim H. S. Kwon J. E. (2017). Sustained Release Docetaxel-Incorporated Lipid Nanoparticles with Improved Pharmacokinetics for Oral and Parenteral Administration. J. Microencapsul. 34 (3), 250–261. 10.1080/02652048.2017.1337247 28557649

[B17] Rizvi S. Z. H. Shah F. A. Khan N. Muhammad I. Ali K. H. Ansari M. M. (2019). Simvastatin-loaded Solid Lipid Nanoparticles for Enhanced Anti-hyperlipidemic Activity in Hyperlipidemia Animal Model. Int. J. Pharm. 560, 136–143. 10.1016/j.ijpharm.2019.02.002 30753932

[B18] Shaveta S. Singh J. Afzal M. Kaur R. Imam S. S. Alruwaili N. K. (2020). Development of Solid Lipid Nanoparticle as Carrier of Pioglitazone for Amplification of Oral Efficacy: Formulation Design Optimization, *In-Vitro* Characterization and *In-Vivo* Biological Evaluation. J. Drug Deliv. Sci. Technol. 57, 101674. 10.1016/j.jddst.2020.101674

[B19] Siekmann B. Westesen K. (1996). Investigations on Solid Lipid Nanoparticles Prepared by Precipitation in O/w Emulsions. Eur. J. Pharm. Biopharm. 42 (2), 104–109.

[B20] Sinha B. Müller R. H. Möschwitzer J. P. (2013). Bottom-up Approaches for Preparing Drug Nanocrystals: Formulations and Factors Affecting Particle Size. Int. J. Pharm. 453 (1), 126–141. 10.1016/j.ijpharm.2013.01.019 23333709

[B21] Smith U. (2001). Pioglitazone: Mechanism of Action. Int. J. Clin. Pract. Suppl. (121), 13–18. 11594239

[B22] Souri E. Jalalizadeh H. Saremi S. (2008). Development and Validation of a Simple and Rapid HPLC Method for Determination of Pioglitazone in Human Plasma and its Application to a Pharmacokinetic Study. J. Chromatogr. Sci. 46 (9), 809–812. 10.1093/chromsci/46.9.809 19007483

[B23] Trevaskis N. L. Kaminskas L. M. Porter C. J. (2015). From Sewer to Saviour - Targeting the Lymphatic System to Promote Drug Exposure and Activity. Nat. Rev. Drug Discov. 14 (11), 781–803. 10.1038/nrd4608 26471369

[B24] Waugh J. Keating G. M. Plosker G. L. Easthope S. Robinson D. M. (2006). Pioglitazone. Drugs 66 (1), 85–109. 10.2165/00003495-200666010-00005 16398569

[B25] Williams H. D. Trevaskis N. L. Charman S. A. Shanker R. M. Charman W. N. Pouton C. W. (2013). Strategies to Address Low Drug Solubility in Discovery and Development. Pharmacol. Rev. 65 (1), 315–499. 10.1124/pr.112.005660 23383426

[B26] Zeb A. Cha J.-H. Noh A. R. Qureshi O. S. Kim K.-W. Choe Y.-H. (2020). Neuroprotective Effects of Carnosine-Loaded Elastic Liposomes in Cerebral Ischemia Rat Model. J. Pharm. Investig. 50 (4), 373–381. 10.1007/s40005-019-00462-y

[B27] Zhang Q. Yang H. Sahito B. Li X. Peng L. Gao X. (2020). Nanostructured Lipid Carriers with Exceptional Gastrointestinal Stability and Inhibition of P-Gp Efflux for Improved Oral Delivery of Tilmicosin. Colloids Surf. B Biointerfaces 187, 110649. 10.1016/j.colsurfb.2019.110649 31767412

